# Progress in Fevers

**Published:** 1902-09-06

**Authors:** 


### Progress in Fevers.

(*Continued from page* 381.)

**Scarlet Fever.**—The diagnosis of scarlet fever is dealt with by J. Marshall Day[10]. The classical symptoms of vomiting, sore throat, rash, elevation of temperature, and increased pulse rate are enumerated. Special stress is laid on the increase of pulse rate, out of proportion to the temperature, which in mild cases may be slight and transient. The presence or absence of complaint of sore throat or dysphagia is delusive; dysphagia may be present in a simple relaxed throat and absent in a severe scarlatinal throat. The fauces should in all cases be carefully inspected. The red punctiform appearance especially on the hard palate is very characteristic. A peculiar dull yellowish white appearance of the hard palate may sometimes be observed in mild cases on the second day. In mild cases the fine punctiform rash should be sought for on the fauces and the soft parts of the skin, the flexures of the limbs, and the sides of the chest and abdomen. It can be detected also on the sides of the fingers and the backs of the hands and feet. A browning of the skin may also be observed in the elbow flexures and at Poupart's ligament, less frequently behind the knees. The "strawberry" tongue, stripped of epithelium and showing prominent fungiform papillæ, must be distinguished from the lightly furred tongue with projecting filiform papillæ observable on the third or fourth day of a gastric catarrh. The milder the case the more delayed the desquamation, which, in very mild cases, may not begin for three weeks and then takes the form of polishing of the finger-tips, roughness at the joint flexures, and later stripping of the feet. In Rötheln vomiting, very common in scarlet fever, is rare, the pulse is less frequent, the fever is slight and commonly subsides in 24 hours, the tongue does not peel, there is no browning at the joint flexures, and glandular enlargement is common. Care is sometimes needed to distinguish scarlet fever from septicæmia or from meningitis. The sudden onset, purplish slightly swelled throat, patchy dark brown or petechial rash, dry tongue, high temperature, and restless semi-conscious state, mark malignant scarlet fever. Rötheln with scarlatiniform rash, "fourth disease," is distinguished by absence of vomiting and rapid pulse and browning of the flexures, presence of enlarged glands, and distribution of the rash all over the face and body. Very strict isolation should be practised in order to stamp out scarlet fever. J. W. Ames[11] suggests, among other things, that all who wish to leave an infected house should do so on the first day of the disease after all their clothes have been disinfected by exposure to formaldehyde gas for six hours. The old idea that the desquamating epithelium of scarlet fever patients is a source of infection is not without challenge.[12] C. K. Millard has received letters from twenty-one authorities on infectious diseases in answer to queries on this subject. Sixteen of these express their belief that the desquamating epithelium is not *per se* a source of infection as has hitherto been firmly held, and that therefore the present lengthened detention of all scarlet-fever patients in hospital may be unnecessary. Dr. Millard is careful to point out that he considers the epithelium shares in the infectivity of other "fomites," but that there is no direct proof, clinical or bacteriological, of the specific virus being contained in the epithelium, as it is believed, for instance, to be contained in the scabs of variolous pustules. Further investigation is, however, desirable. At present the bacteriology of scarlet fever is so little advanced that it would be unwise to disabuse the public of the idea that the peeling skin is infectious. Some success is claimed by Von Leyden[13] and others in treating scarlet fever with injections of serum obtained from patients convalescent from this disease.

**Yellow Fever.**—The contention of E. Souchon[14] that although mosquitos may, yet fomites certainly do, transmit yellow fever is shown by A. H. Doty to rest on insufficient proof,[15] but the views of the latter as to the non-occurrence of secondary cases of yellow fever on board ship are in turn disputed by H. R. Carter.[16] Accepting without reservation the theory of the conveyance of yellow fever by certain infected mosquitos, Dr. Carter maintains that his experience as a quarantine officer shows that a ship can become "infected" by harbouring infected mosquitos—mosquitos which went on board already infected, or which became infected on board by feeding on yellow-fever cases developed on board but contracted on shore. The history of the spread of infection will generally show into which category the mosquitos must fall; nearly always more than two weeks will elapse between the primary and secondary cases if the mosquitos have become infected on board, consequently many vessels will have reached port and have had their infected mosquitos killed by disinfecting processes before secondary cases could have been contracted. If infected mosquitos are shipped, cases may develop at a very short interval after leaving port. Professor Guitéras[17] reports further experiments at Havana on the lines of the U.S.A. Army Commission quite confirming the propagation of the fever by *Stegomyia fasciata*, and the non-infectivity of fomites. He has suggested the immunisation of recently arrived immigrants by inoculation from contaminated insects, having observed the mild cases of fever which resulted from the inoculation experiments of the Army Commission. Only one out of 25 cases in the first series of his experiments developed fever, and seven out of a second series of 17. The character of the attacks is not stated. The mosquitos were infected from very mild cases at a season of the year unfavourable to the disease, and this was thought to explain the many negative results observed. The experiments showed that the mosquito only became infected after biting the patient on the first, second, or third day of the illness, and that at least 12 days must elapse before the inoculated insect becomes capable of transmitting the infection. As a result of the measures taken for the destruction of mosquitos and their breeding grounds, and the screening of yellow fever patients with mosquito-proof wire gauze, Havana was, at the beginning of the year reported free from yellow fever for the first time in 150 years. The sanitary department has worked exclusively on the hypothesis that the Stegomyia mosquito is the sole agent concerned in the propagation of yellow fever.[18] It is, however, to be noted that *Stegomyia fasciata* appears to have no absolute prototype among the sixty odd species of mosquito inhabiting Europe.[19] Yellow fever has at various times appeared in Europe.[20] Several outbreaks have occurred at French and Italian ports. Four times during the last century was the disease brought to Southampton and once to Liverpool.

[10] Dub. Jour. Med. Sci., March 1. [11] Med. Rec., March 22, p. 457. [12] Lancet, April 5. [13] Med. News, May 24, p. 983. [14] Med. Rec., Feb. 8. [15] Ibid., March 8. [16] Ibid., March 22. [17] Practitioner, May, p. 592. [18] Med. Rec., March 22, p. 457. [19] Med. News, June 28, p. 1229. [20] Lancet, July 12, p. 101.

